# A Case of Odontoid Osteomyelitis

**DOI:** 10.7759/cureus.52012

**Published:** 2024-01-10

**Authors:** Etaro Hashimoto, Kenji Miyazaki, Kazuhito Hirose, Tetsuhiro Maeno

**Affiliations:** 1 General Medicine, Mito Kyodo General Hospital, Mito, JPN; 2 General Medicine, Tsukuba Medical Center Hospital, Tsukuba, JPN; 3 Primary Care and Medical Education, Institute of Medicine, University of Tsukuba, Tsukuba, JPN

**Keywords:** calcium pyrophosphate deposition disease, posterior neck pain, staphylococcus aureus infection, spinal epidural abscess, crowned dens syndrome, odontoid osteomyelitis

## Abstract

Odontoid osteomyelitis is a rare infectious disease that manifests as fever and posterior neck pain, while crowned dens syndrome is a relatively common inflammatory disorder with similar signs and symptoms. We describe the case of a 90-year-old woman presenting with fever, posterior neck pain, throat pain, and headache. Crowned dens syndrome was initially diagnosed based on the clinical picture and calcification around the odontoid process on cervical spine CT. However, the diagnosis was revised to odontoid osteomyelitis following the detection of *Staphylococcus aureus* in blood cultures that were performed due to the presence of headache. Infectious complications included spinal epidural abscess extending to the hypoglossal canal and osteomyelitis spreading to the clivus. Nonetheless, the patient achieved complete recovery after 13 weeks of antimicrobial therapy. No reports of odontoid osteomyelitis with calcification around the odontoid process have been reported. This case underscores the importance of avoiding a hasty diagnosis of crowned dens syndrome when calcification around the odontoid process is observed in patients presenting with fever and posterior neck pain. It is crucial to perform a thorough medical history review and physical examination to exclude other conditions. In cases where infection is suspected, blood cultures and cervical spine MRI are essential to investigate odontoid osteomyelitis and other complications.

## Introduction

Odontoid osteomyelitis is a rare infectious disease, usually caused by *Staphylococcus aureus*, that presents with fever and posterior neck pain [1, 2]. Crowned dens syndrome, caused by Calcium Pyrophosphate Deposition Disease (CPDD) in the atlantoaxial joint, presents similarly [3]. In the present case, an older patient with posterior neck pain and fever was initially diagnosed as having crowned dens syndrome. However, based on blood culture results and an MRI scan of the cervical spine, the diagnosis was revised to odontoid osteomyelitis. There have been no reports of odontoid osteomyelitis with calcification around the odontoid process. Since differentiating between odontoid osteomyelitis and crowned dens syndrome based on symptoms alone is difficult, and as this was an instructive case that illustrates the importance of ruling out other diseases, such as infections, before diagnosing crowned dens syndrome, we report the process by which we formulated the final diagnosis.

## Case presentation

**Figure 1:**
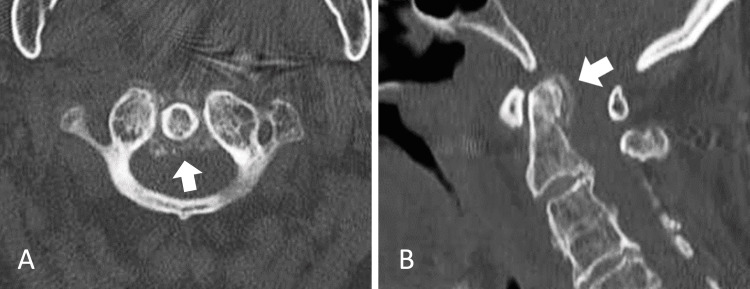
Calcification around the odontoid process on cervical spine CT. A: horizontal section B: sagittal section.

The patient was a 90-year-old woman with a history of C3-C7 vertebroplasty seven years previously. Three days before coming to our hospital, she experienced posterior neck pain, pharyngeal pain, and headache, for which she took acetaminophen. The headache was characterized by pain in all areas of the head, though particularly in the frontal region, and was not associated with nausea. Her symptoms worsened, leading her to request emergency medical assistance and transportation to our hospital. Upon arrival, she was reluctant to move her neck, and her posterior neck pain was most aggravated by rotating her neck from side to side. She did not exhibit rigors but was fully conscious. Her temperature was 38.5°C, blood pressure 142/98 mm Hg, heart rate 94 bpm, respiratory rate 18 breaths/minute, and SpO2 95% on ambient air. The pharynx and tonsils appeared normal. Blood tests revealed a white blood cell count of 12,800/µL and a C-reactive protein level of 15.64 mg/dL. A CT scan of the cervical spine performed without contrast showed calcification around the odontoid process (Figure 1).

**Figure 2:**
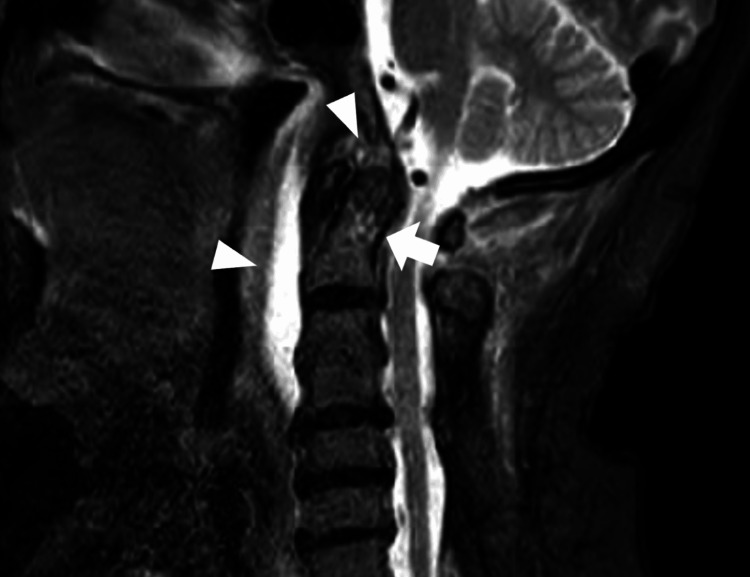
Cervical spine MRI (STIR) showing high signal intensity in the odontoid process (arrow) and fluid accumulation around the odontoid process and anterior to C1-C3 (arrowhead). STIR: Short Tau Inversion Recovery.

**Figure 3:**
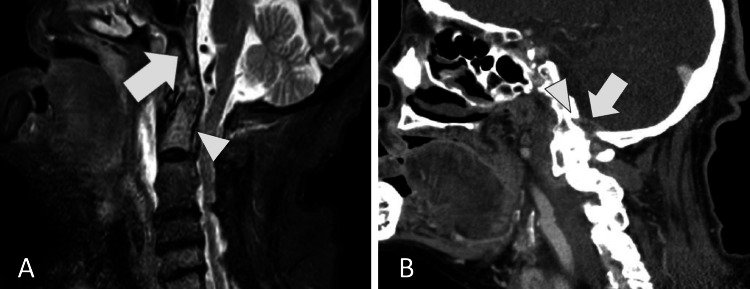
(A) Cervical spine MRI (STIR) with high signal intensity in the odontoid process (arrowhead) and the clivus (arrow). (B) Cervical spine contrast-enhanced CT with abscess (arrow) in the hypoglossal canal (arrowhead). STIR: Short Tau Inversion Recovery.

The posterior neck pain and imaging findings were typical for crowned dens syndrome, but this condition is associated with a relatively low incidence of headache and sore throat [4]. Therefore, considering the possibility of bacteremia, including complications, blood cultures (two sets) were performed, and a nonsteroidal anti-inflammatory drug was prescribed. The next day, *Staphylococci* were isolated from both blood cultures. An additional cervical spine MRI scan using the short tau inversion recovery (STIR) sequence showed high signal intensity in the odontoid process and fluid accumulation around the odontoid and anterior C1-C3 vertebrae (Figure 2), raising the suspicion of odontoid osteomyelitis, spinal epidural abscess, and retropharyngeal abscess. While there were concerns about potential meningitis complications, it was decided not to perform a lumbar puncture because spinal epidural abscess is a contraindication for the procedure due to the risk of bacterial spread into the subarachnoid space [5]. The patient was admitted to the hospital, and an otolaryngologist performed an incision in the posterior wall of the pharynx, but there was no drainage. Empirical treatment was initiated with cefazolin, vancomycin, and piperacillin/tazobactam. Blood cultures soon identified the *Staphylococci* as methicillin-susceptible *S. aureus*. Following bacterial susceptibility testing, de-escalation to cefazolin was implemented. A diagnosis of odontoid osteomyelitis and epidural abscess with calcification around the odontoid process was made. The patient's fever resolved after about a week. An MRI scan of the cervical spine performed two weeks after admission showed that the osteomyelitis had spread to the clivus, and the epidural abscess had extended to the level of the hypoglossal canal (Figure 3).

The spinal cord was not compressed. There was no tongue movement impairment, paralysis of the extremities, or loss of consciousness. The patient's posterior neck pain gradually resolved. A contrast-enhanced CT scan of the thorax and abdomen, a contrast-enhanced MRI scan of the head, and transesophageal echocardiography were performed to look for other foci of infection with *S. aureus*, but none were found. Starting on the sixth day of admission, consecutive blood cultures were negative. During the ninth week of hospitalization, normalization of inflammatory markers was observed in blood tests. We conducted cervical spine CT scans every two weeks and confirmed that fluid retention and bone destruction had not worsened. We administered IV cefazolin for 12 weeks, a duration previously reported to be adequate for osteomyelitis [6], followed by an additional week of oral cephalexin. Following the completion of the treatment, we confirmed the absence of both fever and increased inflammatory markers in blood tests. Consequently, on the 93rd day of hospitalization, the patient was discharged home. There were no sequelae.

## Discussion

We experienced a case of odontoid osteomyelitis with calcification around the odontoid process. Odontoid osteomyelitis is a rare disease, with *S. aureus* being the main causative organism [1, 2]. The primary manifestations include fever and posterior neck pain. Associated complications can include a retropharyngeal abscess, spinal epidural abscess, meningitis, and hypoglossal nerve paralysis [7-9]. Consequently, various signs and symptoms may occur, such as an intense sore throat, limb paralysis, and consciousness impairment. Crowned dens syndrome is a form of CPDD that occurs in the atlantoaxial joint and is common in the elderly [10], characterized primarily by fever and posterior neck pain [3]. Both odontoid osteomyelitis and crowned dens syndrome present with neck pain and marked limitation in neck range of motion, making it difficult to differentiate them by signs and symptoms alone [11]. Nevertheless, pronounced pharyngeal pain may indicate an increased likelihood of odontoid osteomyelitis, particularly when complications such as a retropharyngeal abscess are present [12], while simultaneously reducing the likelihood of crowned dens syndrome [4]. There have been no previous reports of odontoid osteomyelitis with calcification around the odontoid process, as described here, making this combination extremely rare.

In this case, we initially diagnosed crowned dens syndrome based on the typical findings of calcification around the odontoid process on cervical spine CT. However, we also isolated Staphylococcus aureus in blood culture and observed high signal intensity in the odontoid process and fluid accumulation in front of the cervical vertebral body on MRI, none of which are typically associated with crowned dens syndrome. These findings were crucial clues in making the correct diagnosis [1, 7, 9, 13]. When examining a patient with posterior neck pain accompanied by fever and a significantly limited range of motion, it is important to avoid hastily diagnosing crowned dens syndrome based solely on calcification around the odontoid process, as seen on cervical spine CT. Instead, the clinician should actively look for findings inconsistent with crowned dens syndrome, such as intense pharyngeal pain or rigors, and rule out other conditions. If there are signs indicative of an infectious disease, blood cultures should be conducted promptly. Performing an MRI is essential for quickly diagnosing odontoid osteomyelitis and its complications.

## Conclusions

When managing a patient presenting with fever and posterior neck pain, it is risky to immediately diagnose crowned dens syndrome, even if a CT scan reveals calcification around the odontoid process. It is crucial to conduct a comprehensive interview and physical examination to assess the possibility of odontoid osteomyelitis and other cervical diseases based on the patient's signs and symptoms. In cases where infection is suspected, blood cultures and an MRI scan should be performed expeditiously to determine if odontoid osteomyelitis is present.
